# Circulation and seasonality of influenza viruses in different transmission zones in Africa

**DOI:** 10.1186/s12879-022-07727-2

**Published:** 2022-11-07

**Authors:** Marie Roseline Darnycka Belizaire, Anderson Kouabenan N’gattia, Bibata Wassonguema, Marcel Mbeko Simaleko, Emmanuel Nakoune, Clotaire Rafaï, Baidy Lô, Francisco Bolumar

**Affiliations:** 1 School of Medicine, University of Alcalá, Madrid, Spain; 2 National Institute of Public Hygiene, Ministry of Health, Abidjan, Côte d’Ivoire; 3 Inserm, ECEVE, Université Paris Cité, 75010 Paris, France; 4 Faculty of Medical Sciences, University of Bangui, Bangui, Central African Republic; 5 Laboratory for Arboviruses, Viral Hemorrhagic Fevers Viral Hemorrhagic Fevers, Emerging Viruses and Zoonoses, Institut Pasteur de Bangui, Bangui, Central African Republic; 6 National Laboratory and Clinical Analysis, Ministry of Public Health and Population, Bangui, Central African Republic; 7 Faculty of Medicine, University of Nouakchott Al Assriya, Nouakchott, Mauritania; 8 Department of Epidemiology and Biostatistics, Graduate School of Public Health, City University of New York, New York, USA

**Keywords:** Influenza, Africa, Circulation, Seasonality

## Abstract

**Background:**

Influenza is responsible for more than 5 million severe cases and 290,000 to 650,000 deaths every year worldwide. Developing countries account for 99% of influenza deaths in children under 5 years of age. This paper aimed to determine the dynamics of influenza viruses in African transmission areas to identify regional seasonality for appropriate decision-making and the development of regional preparedness and response strategies.

**Methods:**

We used data from the WHO FluMart website collected by National Influenza Centers for seven transmission periods (2013–2019). We calculated weekly proportions of positive influenza cases and determined transmission trends in African countries to determine the seasonality.

**Results:**

From 2013 to 2019, influenza A(H1N1)pdm2009, A(H3N2), and A(H5N1) viruses, as well as influenza B Victoria and Yamagata lineages, circulated in African regions. Influenza A(H1N1)pdm2009 and A(H3N2) highly circulated in northern and southern Africa regions. Influenza activity followed annual and regional variations. In the tropical zone, from eastern to western via the middle regions, influenza activities were marked by the predominance of influenza A subtypes despite the circulation of B lineages. One season was identified for both the southern and northern regions of Africa. In the eastern zone, four influenza seasons were differentiated, and three were differentiated in the western zone.

**Conclusion:**

Circulation dynamics determined five intense influenza activity zones in Africa. In the tropics, influenza virus circulation waves move from the east to the west, while alternative seasons have been identified in northern and southern temperate zones. Health authorities from countries with the same transmission zone, even in the absence of local data based on an established surveillance system, should implement concerted preparedness and control activities, such as vaccination.

## Background

Influenza is an acute and contagious respiratory infection that infects humans and several animal species, including birds and pigs. Outbreaks occur during the winter in temperate regions, and one or two epidemic peaks are sometimes observed in tropical regions. These annual epidemics are responsible for 5 million severe cases and 290,000 to 650,000 deaths worldwide [1]. Approximately 99% of deaths from influenza-associated lower respiratory infections occur in children under 5 years of age in developing countries [2, 3]. To protect the world from the threat of influenza, the WHO established the Global Influenza Surveillance and Response System (GISRS) in 1952. This system operates as a global surveillance, preparedness and response mechanism for monitoring seasonal, pandemic, and zoonotic influenza or novel influenza viruses and other respiratory pathogens [4]. In addition, the WHO recommends that countries develop sentinel surveillance strategies for influenza-like illnesses (ILIs) and severe acute respiratory infections (SARIs) to make better use of the data for public health decisions at all levels of a health system [5]. The purpose of this study was to determine the dynamics of influenza viruses in African transmission zones to identify regional seasonality for appropriate decision-making and the development of regional strategies.

## Methods

### Data collection

Data are provided by the GISRS National Influenza Centers (NICs) and other national laboratories. A total of 148 laboratories from different countries upload weekly data on the FluMart website. Data are publicly accessible online: https://apps.who.int/flumart/Default?ReportNo=12. We selected the following countries using two criteria, including transmission zones and data availability, during the period from 2013–2019: Eastern Africa: Democratic Republic of Congo, Ethiopia, Kenya, Madagascar, Mauritius, Mozambique, Rwanda, Uganda, United Republic of Tanzania, Zambia, and Zimbabwe; Middle Africa: Cameroon and Central African Republic; Western Africa: Burkina Faso, Cote d’Ivoire, Ghana, Mali, Niger, Nigeria, Senegal, and Togo; Southern Africa: South Africa; and Northern Africa: Algeria, Egypt, Morocco, and Tunisia. Variables extracted for the study were country, influenza transmission zone, year, week, the number of specimens processed, the number of influenza A and B viruses detected by subtype and lineage [1, 3, 4, 6].

### Data analysis

This was a descriptive cross-sectional study. The annual proportions of circulating influenza viruses were described by pie charts according to country and transmission zone.

The seasonal transmission dynamics per country and per transmission zone were also described through the definition of mean epidemic curves and alert thresholds. This method used the weekly proportions of influenza-positive cases. First, we identified the weeks with the highest proportions and determined the median peak week for the different years. Then, the weeks with peaks were fitted to the median week. The average of the weekly proportions is reported as the epidemiological curve, the lower bound is reported as the standard deviation, and the upper bound is reported as the mean plus twice the standard deviation. Overlapping epidemic periods by country were grouped to identify seasons in new influenza transmission zones. Data analysis was performed with Stata MP 12 and Tableau Desktop^®^2020.3.3.

## Results

**Fig. 1:**
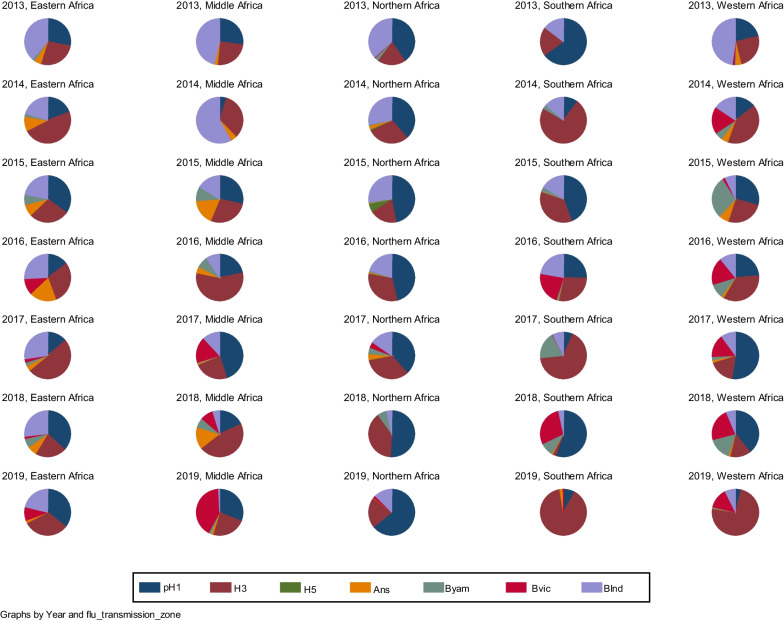
Circulating influenza virus types and subtypes in African influenza transmission areas from 2013 to 2019. Note that pH1 is A(H1N1)pdm2009; H3 is A(H3N2); Ans is A not subtyped; Byam is the B Yamagata virus; Bvic is the B Victoria virus; and Bind is the B virus with undetermined lineage

Figure 1 shows that the circulation of influenza viruses was analyzed by transmission zone over a 7-year period, from 2013 to 2019. Influenza A viruses, in particular A(H1N1)pdm2009, A(H3N2), and A(H5N1), as well as influenza B viruses with the Victoria and Yamagata lineages circulated in the African regions during this period. The influenza activity of the different viruses followed annual and regional variation. In 2013, influenza A viruses predominated in all African regions. However, in terms of lineages and subtypes, the activity of B viruses with undetermined lineages was highest in eastern, middle, northern, and western Africa. In southern and northern Africa, the A(H1N1)pdm2009 virus predominated, where it circulated in high proportions. In 2014, influenza A viruses were supplanted by influenza B viruses in middle Africa. However, influenza A viruses predominated in the other transmission areas. In this context, A(H3N2) supplanted A(H1N1)pdm2009 except in northern Africa. In addition, the B virus with the Victoria lineage has continued to circulate only in western and northern Africa since 2013. In 2015, influenza A viruses predominated over influenza B viruses. A(H1N1)pdm2009 viruses supplanted the A(H3N2) virus in the 5 African transmission areas. The B virus with the Yamagata lineage, which has been circulating only in West Africa since 2014, has reappeared for the first time in southern, eastern, and middle Africa. In 2016, A(H3N2) and A(H1N1)pdm2009 viruses were observed in high proportions in all regions. A(H3N2) was only supplanted by A(H1N1)pdm2009 in northern Africa. The B virus with the Victoria lineage, which has been circulating only in West Africa since 2013, has reached southern and eastern Africa for the first time. In 2017, the A(H3N2) virus predominated in southern and eastern Africa, while the A(H1N1)pdm2009 virus supplanted it in middle, northern, and western Africa. In 2018, A(H1N1)pdm2009 activity continued to predominate in western and northern Africa. It also predominated A(H3N2) in southern and eastern Africa. A(H3N2) has high activity only in middle Africa. In 2019, there was proportional cocirculation of the A(H1N1)pdm2009 and A(H3N2) influenza viruses in eastern and middle Africa. However, in Middle Africa, the B virus with the Victoria lineage predominated all viruses (Fig. 1).

**Fig. 2:**
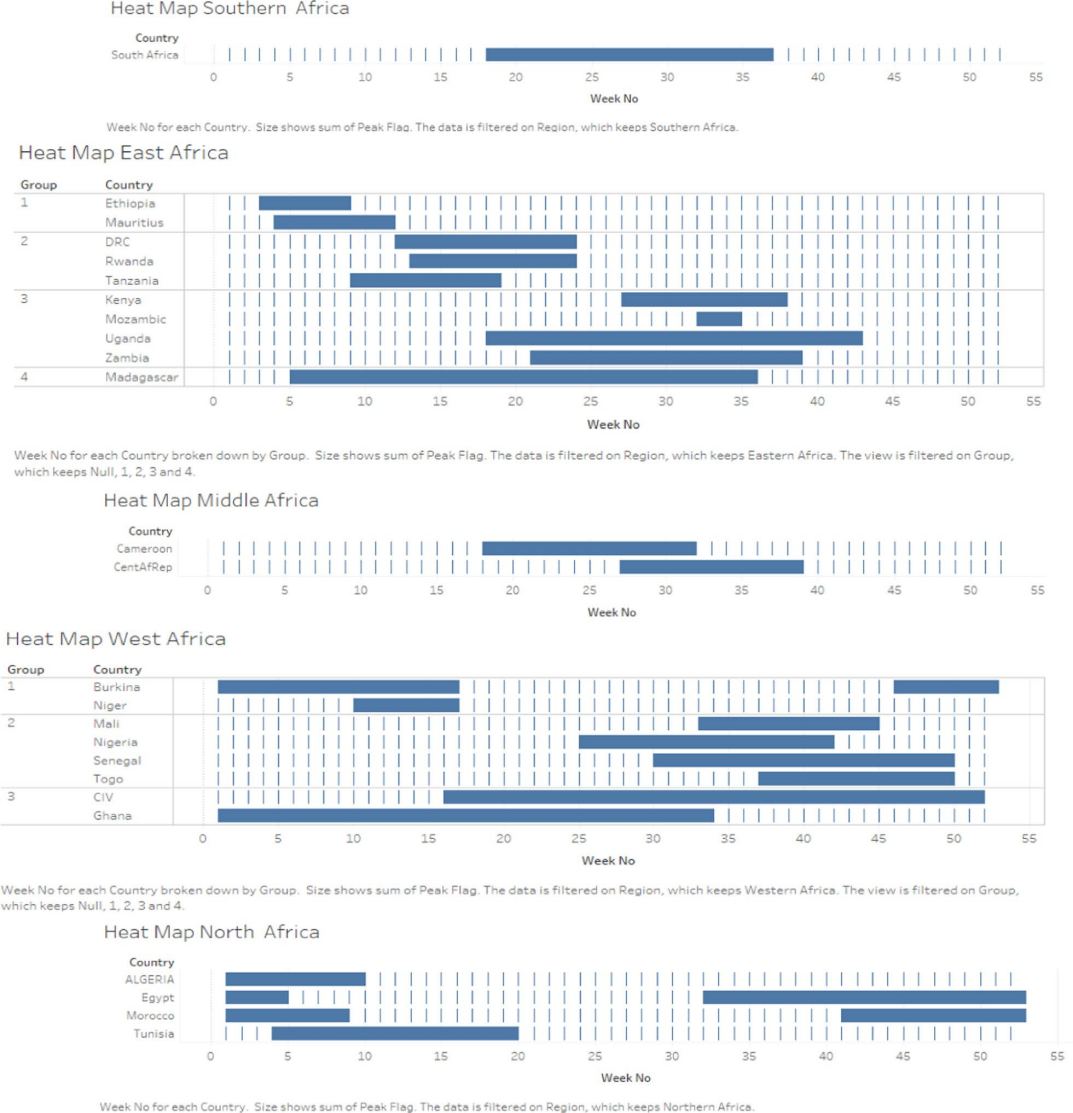
Seasonality of influenza by country and by African transmission zones, 2013–2019. The heatmap by outbreak period in Africa in Fig. 3 shows five periods of high influenza activity

Figure 2 shows a short influenza season running from week 18 to week 37 in southern Africa. In the eastern part of the continent, four influenza seasons are differentiated. The first one occurring from week 3 to week 13 is observed in Ethiopia and Mauritius. The second season occurs from weeks 9 to 15. Countries such as the Democratic Republic of Congo (DRC), Rwanda and Tanzania are subject to the same variation. The third season begins in week 18 and ends in week 43 in Kenya, Mozambique, Uganda, and Zambia. The seasonality of influenza in Madagascar includes the first 3 seasons in the region from week 3 to week 43. Middle African countries, including Cameroon and the Central African Republic, have a single influenza season starting from week 18 to week 39. In western Africa, three influenza seasons were observed. The first one runs from week 44 of the current year to week 20 of the following year. This season occurs in Burkina Faso and Niger. The second season in West Africa runs from week 25 to week 50 in Mali, Nigeria, Senegal, and Togo. In Côte d'Ivoire and Ghana, influenza seasonality occurs between weeks 16 and 52 and between weeks 1 and 34, respectively. The influenza seasons in these two countries are superimposable over weeks 16 to 34. In North Africa, only one influenza season was identified. It starts from week 32 of the current year and continues to week 20 of the following year (Fig. 2).

**Fig. 3:**
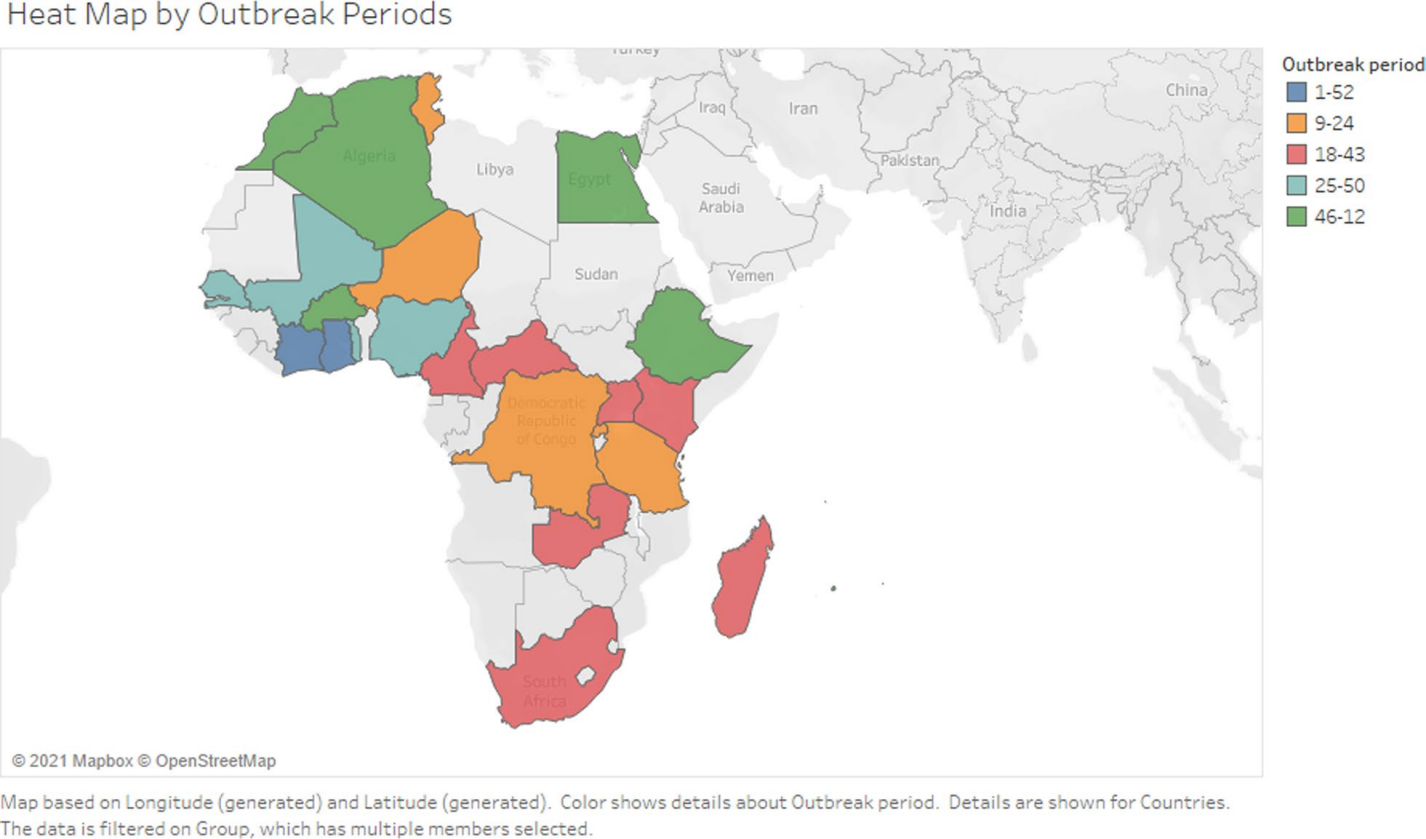
Map of Africa with the table, delimited transmission area, and epidemic period in months in different colors from 2013 to 2019

On Fig. 3, from week 1 to week 52, influenza activity is continuous in countries such as Côte d'Ivoire and Ghana. From weeks 9 to 24, there is a period of high influenza activity in Tunisia, Niger, the DRC, Rwanda, and Tanzania. Cameroon, Kenya, Madagascar, South Africa, Uganda, the Central African Republic, and Zambia are the countries with high influenza activity during weeks 18 to 43. In the period from weeks 25 to 50, influenza epidemic peaks were detected in Nigeria, Mali, Senegal, and Togo. In the period from week 44 of the current year to week 12 of the following year, high influenza activity occurs in the following countries: Morocco, Algeria, Egypt, Ethiopia, and Burkina Faso (Fig. 3).

## Discussion

Influenza virus activity in Africa has followed the global trend. These viruses are the same viruses that have been circulating everywhere else in the world during the same period. These include A(H1N1)pdm2009, A(H3N2), A(H5N1) and influenza B viruses of the Victoria and Yamagata lineages [3, 4]. Indeed, after the 2009 pandemic, only these four influenza viruses continued to circulate on a global scale [5, 7, 8]. Our work has shown that their influenza activity follows an annual and regional variation. The period was mainly marked by a high activity of the A(H1N1)pdm2009 and A(H3N2) viruses in the temperate zones of North and South Africa, while influenza B viruses showed a high activity in the African tropical belt. Our results were confirmed by other studies. These studies revealed high proportions of seasonal influenza A activity in temperate countries versus high rates of influenza B activity in the sub-Saharan region [9, 10]. In addition to the fact that influenza seasons have always been dominated by A(H1N1)pdm2009 or A(H3N2) in the temperate zone of southern Africa and by A(H1N1)pdm2009 in northern Africa, our work has shown that in the tropical African belt (East, West and Middle), the B viruses that were in high circulation in 2013 were progressively replaced by A(H3N2) and then replaced a few times by A(H1N1)pdm2009 beginning in 2014. However, the B virus with the Victoria lineage, isolated in the 2013 season in Western and Northern Africa, continued to grow in proportion in Middle Africa until it predominated all others in the 2019 season. For this reason, some authors have stated that the influenza activity of type B viruses is relatively strong in the tropics, but it has often been predominated by that of type A viruses, which is more intense and longer lasting [5, 8, 10, 11].

The data from the study show that in southern Africa, the influenza epidemic seasons occur from May to August, corresponding to southern winter. In the tropical part of the continent, influenza seasons start in the eastern region and extend to the western region, lasting almost the whole year. One and even four epidemic peaks could be identified depending on the area of transmission. However, exceptions were observed in western Africa, where the Sahelian zone (Group 1) followed a seasonality similar to that observed in the northern temperate countries. In fact, in Northern Africa, the impact of the cold Mediterranean climate results in an influenza season that extends from August to April. Similarly, Alonso et al. showed through a global map that the influenza circulation patterns in temperate countries were well aligned with the winter seasons for both the Northern and Southern Hemispheres and the running seasons for tropical countries from February to November [4]. The observed circulation patterns have been demonstrated to be driven by certain climatic factors, including temperature for temperate regions and rain, humidity, and drought for tropical regions [5, 12–14].

Globally, in Africa, five influenza seasons are identifiable in the five transmission zones. The influenza season, which runs from March to October, extends from the southern African transmission zone to the middle and eastern African transmission zones. At these times, influenza activity is strongly influenced by climatic effects [15]. In the transmission areas of North and West Africa, especially near the coast, the influenza seasons occur somewhat later and are spread throughout the year. These late winter activities of influenza viruses in these transmission areas sometimes coincide with climatic phenomena such as cold, *el-niño* and *la niña* [5, 13, 16, 17]. However, exceptionally for Madagascar, influenza seasons are spread throughout the year. This study corroborates the conclusions of Alonso et al. that in Madagascar, influenza activity is irregular and not influenced by the climate [18]. Furthermore, WHO vaccination timing and formulation recommendations suggested that the Northern Hemisphere vaccine be provided for immunization in October for Mali and Madagascar [19]. Conversely, our results suggested that the Southern Hemisphere vaccine be provided in April for both countries, even though Guillebaud et al. assumed that routine influenza vaccines (Northern and Southern hemispheres) would not provide optimal protection against half of the influenza strains circulating in any epidemic season of Madagascar [20]. Additionally, the WHO recommended that Cote d’Ivoire and Ghana start immunization in April with the Southern Hemisphere vaccine (OMS). However, our findings demonstrated the year-round circulation of influenza in these countries. This assumption and the findings of Guillebaud should advise the WHO to closely tailor routine influenza immunization to local patterns of viral circulation rather than a country’s hemispheric position [4].

Prevention, preparedness, and response measures should be implemented prior to the influenza circulation patterns in each transmission area, including from January to December for West Africa, February to December for East Africa, February to October for Middle and South Africa and November to March for North Africa.

Our study is limited by the inclusion of data exclusively from the WHO FluMart website. Despite the relevance and quality of this free and open access data, it may be different in different countries. However, this may not have influenced the quality of our work thanks to the multitude of data from the included countries.

## Conclusions

Seven years of data from the GISRS network showed that the influenza viruses circulating in the five transmission zones in Africa are similar to those isolated elsewhere in the world. In this period, influenza A(H1N1)pdm2009 and A(H3N2) had strong activity in northern and southern Africa, respectively, while influenza B viruses, mainly the B virus with the Victoria lineage, showed high activity in middle Africa.

Their activity generates different epidemic periods in the transmission zones each year. In southern Africa, a single epidemic season extends from May to August, while in the northern zone, it extends from August to April across 2 years. It has also been shown that in the tropical belt of the continent, influenza seasons start in the east and end in the west. They generate three main epidemic peaks in the East, one in the Middle and three in the West. Health authorities in different transmission zones need to implement concerted preparedness and control activities before the epidemic periods. Despite the lack of surveillance systems in some countries, the synchronization of preparedness and control activities should include all countries from the transmission zones to limit the impact of influenza on their populations.

## Data Availability

The datasets generated and/or analyzed during the current study are available in the GISRS repository, [https://apps.who.int/flumart/Default?ReportNo=10].

## Abbreviations

WHO: World Health Organization
GISRS: WHO Global Influenza Surveillance and Response System
NIC: National Influenza Center
pH1: A(H1N1)pdm09
H3: A(H3N2)
Ans: Influenza A not subtyped
Byam: Influenza B Yamagata
Bvic: Influenza B Victoria
Bind: Influenza B with undetermined lineage
DRC: Democratic Republic of Congo
